# 17-Week Delay Surgery after Chemoradiation in Rectal Cancer with Complete Pathological Response

**DOI:** 10.1155/2015/816491

**Published:** 2015-10-22

**Authors:** Marisa D. Santos, Manuel T. Gomes, Filipa Moreno, Anabela Rocha, Carlos Lopes

**Affiliations:** ^1^Department of Surgery, Digestive Surgery Service, Hospital de Santo António, Centro Hospitalar do Porto, Largo Professor Abel Salazar, 4099-003 Porto, Portugal; ^2^Department of Medical Imaging, Radiology Service, Hospital de Santo António, Centro Hospitalar do Porto, Largo Professor Abel Salazar, 4099-003 Porto, Portugal; ^3^Department of Pathology, Pathological Anatomy Service, Hospital de Santo António, Centro Hospitalar do Porto, Largo Professor Abel Salazar, 4099-003 Porto, Portugal; ^4^Department of Pathology and Molecular Immunology, Instituto de Ciências Biomédicas Abel Salazar, Rua Jorge Viterbo Ferreira No. 228, 4050-313 Porto, Portugal

## Abstract

Neoadjuvant chemoradiation (CRT) followed by curative surgery still remains the standard of care for locally advanced rectal cancer (LARC). The main purpose of this multimodal treatment is to achieve a complete pathological tumor response (ypCR), with better survival. The surgery delay after CRT completion seems to increase tumor response and ypCR rate. Usually, time intervals range from 8 to 12 weeks, but the maximum tumor regression may not be seen in rectal adenocarcinomas until several months after CRT. About this issue, we report a case of a 52-year-old man with LARC treated with neoadjuvant CRT who developed, one month after RT completion, an acute myocardial infarction. The need to increase the interval between CRT and surgery for 17 weeks allowed a curative surgery without morbidity and an unexpected complete tumor response in the resected specimen (given the parameters presented in pelvic magnetic resonance imaging (MRI) performed 11 weeks after radiotherapy completion).

## 1. Introduction

Neoadjuvant chemoradiation (CRT) followed by total mesorectum excision (TME) surgery and systemic chemotherapy still remains the standard of care for locally advanced rectal cancer (LARC), but not all cases benefit from this treatment modality. This multimodal treatment improves disease pelvic control but better survival is achieved only if pathological response is present [1, 2]. In LARC, neoadjuvant CRT is extensively used to decrease the risk of local failure by sterilizing microscopic tumor foci not removed by the surgeon. It also allows radical surgery in difficult resectable tumors and increases the chances of sphincter-saving surgery. For a successful outcome, the size or the stage of the rectal tumor must be decreased. An interval between the end of CRT and surgery is required for tumor regression. Moreover, tumor regression varies between complete, poor, and even absence response. Tumor regression grade depends on tumor biology, CRT scheme, and the time interval between the end of CRT and surgery. If tumor response is complete or near complete, we have a favorable tumor biological profile with less risk of recurrence and better survival. When tumor CRT response is present, prolonging the time interval between CRT and surgery in order to achieve maximal tumor regression and diminish complications during surgery is advisable. On the other hand, if the tumor is a poor responder, a longer time interval after CRT is counterproductive. Maximum tumor regression may not be seen in rectal adenocarcinomas until after several months after the end of CRT; thus, a longer than usual CRT delay may benefit those well responding tumors. Predicting tumor response is not possible yet and the delay between neo-CRT and surgery in most studies ranges between 8 and 12 weeks. The optimal interval time between neoadjuvant CRT and surgery for rectal cancer has been debated.

In this regard we report a patient with LARC where the need to increase the interval between neo-CRT and surgery in 17 weeks was safe and allowed a complete tumor response in the resected specimen that was not present in the pelvic MRI performed 11 weeks after radiotherapy completion.

## 2. Case Report

**Figure 1:**
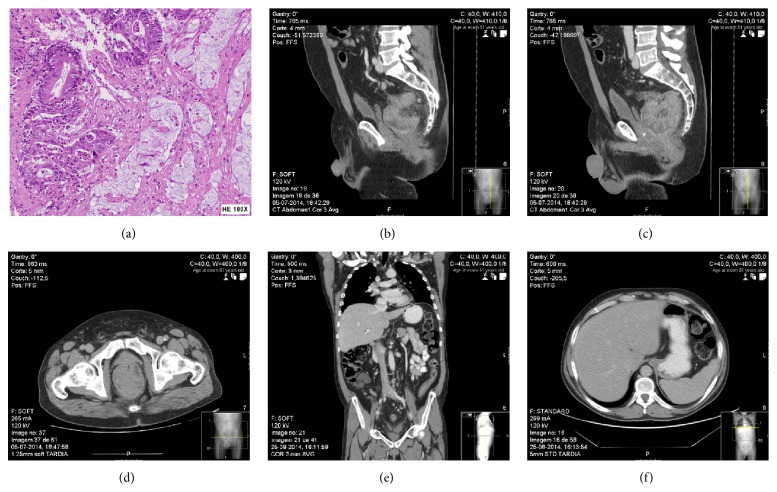
Diagnosis and staging. (a) Rectal biopsy mucinous adenocarcinoma; (b), (c), (d), (e), and (f) CT scan large tumor with 9 cm of longitudinal length and 7 cm of transverse diameter, plan between tumor and prostate not evident, multiple enlarged pelvic lymph nodes, and no evidence of inguinal nodal involvement or distant metastasis.

A 52-year-old man, with a 4-month history of bloody stool, change in size and shape of stools, and feeling of incomplete defecation, is subjected to a colonoscopy that revealed ulcer vegetative circumferential tumor at 6 cm of anal margin not crossable with colonoscope. Biopsy resulted in ulcerative adenocarcinoma (Figure 1(a)).

**Figure 2:**
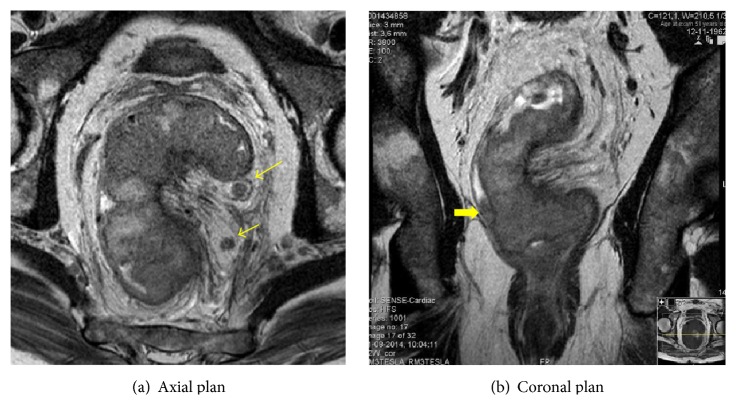
MRI before CRT. Large tumor with 9 cm of longitudinal length and 7 cm of transverse diameter that extends to left side of mesorectal fascia, multiple enlarged pelvic lymph nodes. Thin arrow: metastatic lymph node; thick arrow: mesorectal fascia invasion.

Physical examination confirmed ulcerative circular lesion, fixed with lower edge at 6 cm of anal margin, without enlarged inguinal lymph nodes. CT scan (Figures 1(b), 1(c), and 1(d)) and MRI (Figures 2(a), 2(b), and 2(c)) revealed a large tumor with 9 cm of longitudinal length and 7 cm of transverse diameter that extended to left side of mesorectal fascia, multiple enlarged pelvic lymph nodes, and no evidence of inguinal nodal involvement or distant metastasis.

**Figure 3:**
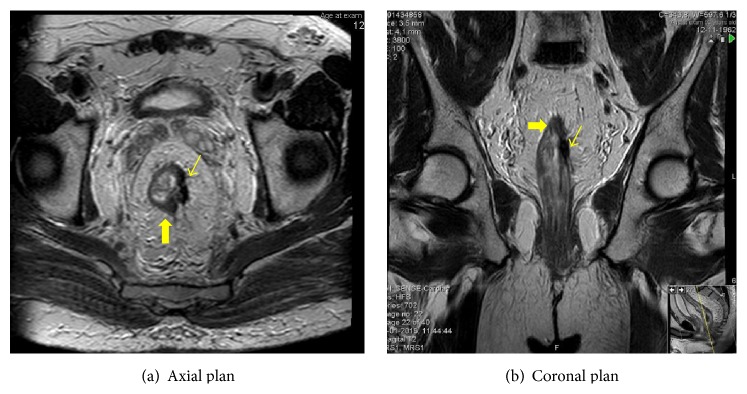
MRI 11 weeks after CRT completion. Good tumor response: tumor downsizing and downstaging, nodal downstaging, and increase between tumor and mesorectal fascia. Thin arrow: areas of fibrosis (very low signal intensity); thick arrow: areas of residual tumor (intermediate signal intensity).

Tumor staging was T4N+M0. Laboratory data showed a normal carcinoembryonic antigen serum level. Before initiating therapy, a laparoscopic colostomy was performed to avoid bowel obstruction. Neoadjuvant chemoradiotherapy was given: a total irradiation of 50.4 Gy in 28 fractions and capecitabine 825 mg/m² (five weeks). CRT progressed uneventfully but, four weeks after the end of RT, the patient had an acute myocardial infarction requiring coronary stents and dual antiplatelet therapy. For this reason, surgery planned for 8 weeks after the end of RT was delayed. In the meantime, 11 weeks after RT completion a restaging MRI was performed. The size of rectal tumor had dramatically diminished, with tumor downstaging and increased mesorectal distance. Nonetheless, there were still some small nodular areas at the periphery of the posterior margin of the rectum that were suspicious for residual tumor (Figures 3(a), 3(b), and 3(c)).

**Figure 4:**
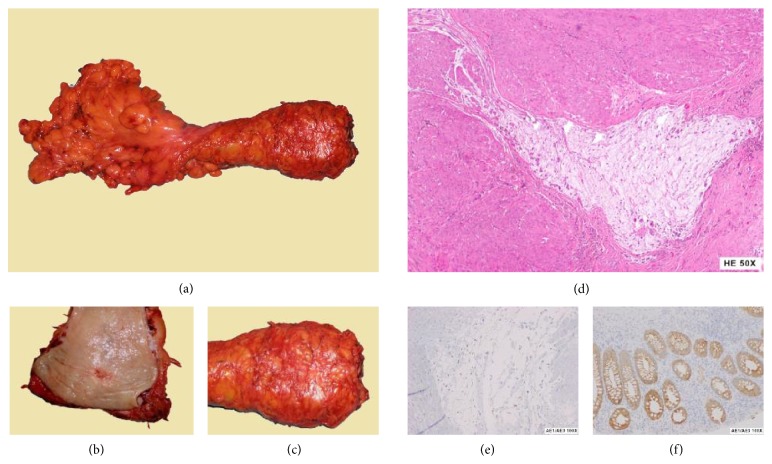
Resected specimen and histological result. (a) Resected specimen; (b) and (c) details of resected specimen; (d) mucin pools with histiocytes in muscular layer but no malignant epithelium; (e) and (f) immunohistochemistry analysis with cytokeratin AE1/AE3 confirms the absence of malignant epithelium.

17 weeks after radiotherapy the patient cardiac function recovered, and a radical surgical resection was performed. A conventional ultralow anterior rectal resection, with total mesorectum excision, was performed after colostomy closure by open access (Figures 4(a), 4(b), and 4(c)).

Bowel transit reestablishment was carried out by a coloanal end-to-end anastomosis. A diverting ileostomy was placed in the previous colostomy site. The postoperative course was uneventful and the patient was discharged from hospital 7 days after surgery. The resected specimen revealed mucin pools but no malignant epithelium, complete pathological response. In order to ensure the absence of epithelial cells within the mucin pools, the entire area containing macroscopic alteration was submitted to histopathological and immunohistochemistry analysis (Figures 4(c) and 4(d)). The ileostomy closure was performed 6 weeks after rectal surgery.

## 3. Discussion

The achievement of ypCR is the ultimate goal of LARC therapeutic with neoadjuvant RCT. When present, ypCR implies a better survival and delaying radical surgery and adopting a “wait and see” strategy might be appropriate when the patient is unfit because of comorbidities or is unwilling to undergo radical surgery.

Two major problems remain: rates of low ypCR and difficulty in defining clinical complete response (cCR) at restaging MRI, after completion of CRT.

Only ypCR rates of 13–30% have been reported in phase II and phase III trials following 5FU based preoperative CRT. A way to increase this rate is delaying the interval between neoadjuvant CRT and surgery. The optimal interval remains debatable. In 1999, Francois et al. [3] compared short (within 2 weeks) and long (6 to 8 weeks) interval groups following preoperative radiotherapy (RT) and showed that longer intervals were associated with better clinical tumor response, pathological downstaging, and a nonsignificant trend towards increased sphincter preservation. They suggested that a longer interval may increase the chance of successful sphincter-saving surgery through improved tumor response. The increased intervals could potentially increase the tumor downstaging effect as the radiation-induced necrosis appears to be a time-dependent phenomenon. As a consequence, this 6- to 8-week interval has become part of the standard protocol for the treatment of mid and low rectal cancer. Recently, several studies including a meta-analysis suggested that this interval can be further expanded (8- to 12-week) with higher rates of pCR and downstaging, decreased recurrence, and improved DFS [4–6]. Based on these studies, in last 12 years, our institution adopted the mean interval of 8 weeks without more anastomotic complications [7]. Intervals longer than 12 weeks are not recommended. There is a potential risk of emergence of subclinical tumors which can grow more rapidly than the primary tumor and increase risk of developing distant metastases [8, 9]. Also, fibrosis that could be established may lead to surgical technical difficulties, with increased morbidity.

Emerging evidence has shown the prognostic importance of reassessing rectal cancers using high-resolution T2-weighted MRI after completion of CRT [10–12]. These reassessment MRIs have implications in surgical planning, timing of surgery, sphincter preservation, deferral of surgery for good responders, and development of further preoperative treatments for imaging poor responders. It should be stressed however that MRI findings depend on the ability to differentiate the tumor from posttreatment fibrosis besides the MRI timing. On post-CRT T2-weighted MR imaging, we found that areas of fibrosis have very low signal intensity, whereas areas of residual tumor have intermediate signal intensity, which can be difficult to interpret. In the last two years, our institution performed MRI in LARC treated with neoadjuvant CRT restaging, 6 weeks after CRT completion.

In our case report, posttreatment MRI was realized 11 weeks after CRT completion and revealed a good response to treatment, but not a complete response. Post-CRT T2-weighted MRI showed areas of intermediate signal intensity that were interpreted as residual tumor. Delaying surgery for 17 weeks afforded an unexpected complete pathological response: tumor response was prolonged beyond 11 weeks. On the other hand, contrary to expectations, this did not lead to an increased difficulty in surgery or an increased morbidity.

Delaying surgery more than 12 weeks in patients with good response to CRT does not seem to have a negative impact on patients prognosis: this is valid in ypCR, in accordance with “wait and see policy” [13, 14] but must be confirmed in patients with partial response to CRT.
